# Supplementary material for: Epidemiology of soil-transmitted helminthiasis among school-aged children in pastoralist communities of Kenya: A cross-sectional study
Source: PLoS One. 2024 May 23;19(5):e0304266. doi: 10.1371/journal.pone.0304266 (PMC11115206; doi:10.1371/journal.pone.0304266)
Supplement: S1 Table — (DOCX) [file pone.0304266.s001.docx]

**S1 Table: Multivariable analysis of risk factors associated with A. lumbricoides infections**

| **Factors** | **Adjusted Odds Ratio [aOR(95%CI)]** | **p-value** |
| --- | --- | --- |
| **School** |  |  |
| Karda | 3.74 (0.80-17.47) | 0.093 |
| Nkarano | 7.81 (1.81-33.63) | 0.006* |
| Olereko | 0.73 (0.11-4.95) | 0.743 |
| Olookwaya | 0.46 (0.04-4.93) | 0.518 |
| Pusanki | Reference |  |
| **Household members** |  |  |
| No. of adults |  |  |
| <5 | Reference |  |
| 5-10 | 1.04 (0.23-4.79) | 0.956 |
| >10 | - |  |
| **Water source for drinking and cooking** |  |  |
| Rainwater |  |  |
| Yes | 2.84 (0.69-11.71) | 0.149 |
| No | Reference |  |
| **Type of house material** |  |  |
| Mud | Reference |  |
| Cement | 0.39 (0.09-1.63) | 0.198 |
| Wood | 1.47 (0.10-21.38) | 0.778 |
| Others | 1.30 (0.10-17.40) | 0.842 |

*significant variables at p<0.05
