# Supplementary material for: Epidemiology of soil-transmitted helminthiasis among school-aged children in pastoralist communities of Kenya: A cross-sectional study
Source: PLoS One. 2024 May 23;19(5):e0304266. doi: 10.1371/journal.pone.0304266 (PMC11115206; doi:10.1371/journal.pone.0304266)
Supplement: S2 Table — (DOCX) [file pone.0304266.s002.docx]

**S2 Table: Multivariable analysis of risk factors associated with T. trichiura infections**

| **Factors** | **Adjusted Odds Ratio [aOR(95%CI)]** | **p-value** |
| --- | --- | --- |
| **Household members** |  |  |
| No. of children |  |  |
| <5 | Reference |  |
| 5 - 10 | 2.00 (0.57-7.01) | 0.279 |
| > 10 | 3.52 (0.63-19.57) | 0.151 |
| Family occupation |  |  |
| Employed |  |  |
| Yes | 2.71 (0.88-8.36) | 0.083 |
| No | Reference |  |
| **Water source for drinking and cooking** |  |  |
| Borehole or well |  |  |
| Yes | 1.30 (0.26-6.42) | 0.745 |
| No | Reference |  |
| **Type of activities involved with at home** |  |  |
| Cooking |  |  |
| Yes | 0.29 (0.06-1.34) | 0.113 |
| No | Reference |  |
| **Frequency of shoe wearing** |  |  |
| Always | 0.37 (0.11-1.22) | 0.103 |
| Occasionally | Reference |  |
