# Supplementary material for: Epidemiology of soil-transmitted helminthiasis among school-aged children in pastoralist communities of Kenya: A cross-sectional study
Source: PLoS One. 2024 May 23;19(5):e0304266. doi: 10.1371/journal.pone.0304266 (PMC11115206; doi:10.1371/journal.pone.0304266)
Supplement: Attachment — Submitted filename: Comments.docx [file pone.0304266.s003.docx]

**Epidemiology of soil -transmitted helminthiasis infection among school aged children in pastoralist communities of Narok County, Kenya: A cross-sectional study**

**Comments**

Line 23 – add County after word Narok

Line 31 – 37 I find it encouraging to find that the data indicate hookworm prevalence is zero. Did the study seek or want to share the possible causes of the success stories from the ground to be emulated to control the other STH infections eg **Trichuris trichiura**?

This area of study is known to have a close interface between human, livestock and wildlife animals. It would probably be good to enrich discussion on this sphere. This perspective is important probably while designing control strategy

Line 39 – 44 The conclusion that there should be improvement of the WASH in selected schools is limiting. I suppose that the infections could not only be attributable to the schools alone. It could be possible that there is lapse of sanitation in homes/associated environment and thus acting as disease reservoir(s). The recommendation in my opinion would be to improve WASH holistically both in the school and the associated catchment area although the areas may not have been investigated in the current study.

Line 81 – I suggest replacement of the word country with Kenya so that it can be more specific.

Line 97 – First word should be Dominant not dominate.

Line 145 - Parasitological examination

It would be good to provide details of: - what power was used for microscopy examination, how the values were translated to eggs per grams and reference the classifications of egg intensity if it was of interest in the study. Was there random examination of the slides for QC/QA especially for the purposes of infection intensity accuracy?

**Study design**

Although of sampling schools was purposive, how did the study ensure minimal bias and put consideration of equal representation of areas? This would enable all areas to be represented and prevent over sampling of the possible disease hotspots?

**Results**

Line 174 – Why was this school - Olookwaya 86 (16.7%) under sampled in terms of participants compared to the rest of the schools. Interestingly, the school had the highest prevalence of *Trichuris trichiura* which also comprises the largest burden of the specific STH in the study. Is it by design or an oversight?

Line 213 - Mean intensity of the infections

Would it be possible to express this in reference to WHO classifications?

**Conclusions**

Line 383 ­– the conclusion proposes use of drug combination that are effective for use to control infection but ran short of mentioning the recommended components or what should be included/added to the current therapy? Any leads? Make the recommendation more specific!

According to this study, is there need for incorporating environmental based interventions as a complementary strategy? Just a thought

**Verdict:** The manuscript should be published once the few comments are addressed.
