# Supplementary material for: Epidemiology of soil-transmitted helminthiasis among school-aged children in pastoralist communities of Kenya: A cross-sectional study
Source: PLoS One. 2024 May 23;19(5):e0304266. doi: 10.1371/journal.pone.0304266 (PMC11115206; doi:10.1371/journal.pone.0304266)
Supplement: Attachment — Submitted filename: Response to Reviewers_06052024.docx [file pone.0304266.s005.docx]

Emmannuel Timmy Donkoh, PhD

Academic Editor

PLOS ONE

6^th^ May 2024

Dear Dr. Donkoh,

Re: Revised version of manuscript reference PONE-D-23-39548

Thank you for the opportunity to revise our manuscript titled "Epidemiology of soil-transmitted helminthiasis among school-aged children in pastoralist communities of Kenya: A cross-sectional study" for submission to PLOS ONE. We are grateful for the valuable feedback from both you and the reviewers, which has helped enhance our paper. We are pleased to resubmit the article for your further consideration, incorporating changes based on your insightful suggestions. We believe our revisions and the responses provided below adequately address the issues and concerns raised

For your review here is a point by point response to the comments and questions raised. The comments are indicated in bold while our responses are not bold.

**Journal Requirements**

1. **Please review your reference list to ensure that it is complete and correct**

We have gone through the reference list and have ensured that all the cited papers are complete and correct and no cited paper has been retracted. From the initial submission I added five references which have been listed appropriately as reference number 29, 38, 39, 41 and 42 in the reference list. The complete reference list can be found from Line 490 to 663.

**Additional Editor Comments**

1. **Comment on the title to be specific, descriptive, concise and comprehensible as per journal requirements**

Thank you for this comment. I have revised the title accordingly to reflect the journal requirements as "Epidemiology of soil-transmitted helminthiasis among school-aged children in pastoralist communities of Kenya: A cross-sectional study"

**Outstanding Reviewer 1 Comment**

1. **The point I raised as #4 in my initial review has not been addressed by the authors.
   “4. since the school seems to be the most important factor for infection prevalence, the authors should better elaborate about the possibility that some of the school reached low MDA coverage (for example if the schools are more difficult to access or there reduced school attendance or again there is less compliance among the teachers or children” I understand the coverage data are not available at school level and therefore it is not possible to assess if the differences in STH prevalence measured at school level can be due to difference in coverage. I suggest however to mention this in the limitation chapter of the manuscript.**

Thank you for this comment, indeed it is a limitation of the study as MDA coverage was reported at the sub-county level rather than the school level, due to data availability constraints. Thus, some schools may have experienced low MDA coverage due to factors such as accessibility, reduced school attendance or compliance among teachers or children. This has been explained in Line 443 to 447 of the limitations section of manuscript

Authors wish the version will satisfy the editor and reviewers and meet all standards of PLOS ONE. The authors welcome further comments, if any.

Yours sincerely,

Richelle W. Kihoro

Corresponding author
